# Rush Medical College

**Published:** 1849

**Authors:** 


### ARTICLE VIII.

### RUSH MEDICAL COLLEGE.

It will be seen by the notice on the cover, that the Trustees, of this Institution, are disposed to spare no pains in their endeavours to fill the two vacant chairs, with the best talent at their command. We are satisfied that they will be governed entirely by the interest of the College in making the appointments. To enable them to act understandingly, applicants would do well to send evidence of character, qualifications, and experience in practice and teaching.

They think justly that the flattering prospects of the school and the advantages offered by the City of Chicago as a location for one holding a chair in it, ought to bring into competition for these places the ablest men in the profession.

For the information of physicians at a distance, we would state that the College was organized six years ago, under an independent charter from the Legislature of Illinois, to the board of Trustees, and has been in successful operation ever since. The last class numbered 100 Students, and would have been one third larger had the same terms of credit been given as last year, and as neighboring schools offered.

The chairs are on an equality so far as regards the price of tickets, and a voice in the management of buisness are concerned.

A commodious brick building, large enough to accommodate 200 students.; situated in a convenient part of the City, belongs to the Institution.

There is an abundance of material for dissection always at command, and plenty of dispensary and hospital practice affording means of giving Clinical instruction.

The City of Chicago numbers over 20,000 inhabitants, which has for several years been increasing at about 20 per cent. per annum, and from its position and prospects, will no doubt be the great commercial city of the North-West. E.

